# Enzymatic Characterization of Purified *β*-Glucosidase from Non-*Saccharomyces* Yeasts and Application on Chardonnay Aging

**DOI:** 10.3390/foods11060852

**Published:** 2022-03-17

**Authors:** Pingping Gao, Faisal Eudes Sam, Bo Zhang, Shuai Peng, Min Li, Jing Wang

**Affiliations:** 1 College of Food Science and Engineering, Gansu Agricultural University, Lanzhou 730070, China; gaoping2634@163.com (P.G.); sameudes0@gmail.com (F.E.S.); zhangbo@gsau.edu.cn (B.Z.); pengs@gsau.edu.cn (S.P.); limin@gsau.edu.cn (M.L.); 2 Gansu Key Lab of Viticulture and Enology, Lanzhou 730070, China

**Keywords:** *Hanseniaspora uvarum*, *Meyerozyma guilliermondii*, *β*-glucosidase, enzymatic characterization, aging, volatiles

## Abstract

The application of *β*-glucosidase from non-*Saccharomyces* yeasts to improve wine aroma has been widely explored. However, few enzymes are active under the severe conditions of wine aging (high ethanol concentration, low temperature, and low pH). Therefore, the application of *β*-glucosidase in wine aging needs further research. In this study, the *β*-glucosidases Mg-βgl and Hu-βgl extracted from *Meyerozyma guilliermondii* NM218 and *Hanseniaspora uvarum* BF345 were purified and used in young Chardonnay wines aged for 50 days. The enzyme activity of the two enzymes was measured. The effects of the two enzymes and a commercial *β*-glucosidase (An-βgl) on the volatile composition and sensory quality of the wine were also determined. The results showed that Mg-βgl and Hu-βgl had high specific activity of 1.95 U/mg and 2.11 U/mg, respectively, maintaining the activity of 70–80% at 20 °C, pH of 3.0–4.0, and 15% ethanol, corresponding to wine aging conditions. Analysis of volatiles with GC-MS showed a 65–70% increase in total terpenoids and new detection of C_13_-norisoprenoids when the wines were treated with the three *β*-glucosidases. In addition, wines treated with Mg-βgl and Hu-βgl had more hexanol, phenylethanol, ethyl octanoate, ethyl heptanoate, and ethyl caprate than wines treated without and with An-βgl. In sensory analysis, the judges showed a greater preference for Hu-βgl-treated wines, to which they attributed pleasant sweet, floral, honey, pomelo, and banana aromas. The results of this study not only offer a way to improve flavor complexity in wine but also provide a reference for the use of other edible sources of *β*-glucosidase in wine aging.

## 1. Introduction

Volatile aroma compounds are a vital component of wine quality, with more than eight hundred being identified in wine, including monoterpenes, aldehydes, ketones, esters, organic acids, and alcohols [1,2]. These compounds, which are derived from grapes, fermentation, or post-fermentation treatments such as oak storage and bottle aging, are found in must and wines mostly in the form of glycosidic conjugates [3]. Glycosidase enzymes are effective tools for the release of aroma compounds due to their ability to break the bond-linkage between a sugar moiety and an aglycone [4]. A large portion of glycosides remains in young wines as a result of the limited impact of glycosidases from grape and *Saccharomyces cerevisiae* in winemaking [5]. Therefore, the use of *β*-glucosidases would be of interest to wine producers and wineries seeking to improve wine aroma quality since they can promote the liberation of aroma compounds from monoterpene glycoside precursors present in young wines [6,7].

Most enzymes used in winemaking are derived from fungi, such as *Aspergillus niger*, and a non-specific mixture of enzymes that contain multiple enzymatic activities [8], which are commonly used by some wineries to enhance the release of aroma compounds [9]. However, such enzymes may produce unpredictable adverse reactions [8]. Some studies have shown that the continuous use of these commercial enzymes can significantly reduce the aroma complexity and alcoholic strength of wines [10,11]. Alternatively, other reports have highlighted the *β*-glucosidase producing potential of non-*Saccharomyces* yeasts [12]. Several literature reports have shown that some *β*-glucosidases from non-*Saccharomyces* yeasts could function at low pH, low temperature, and high ethanol concentrations [13]. However, the activity of *β*-glucosidases in grape must and wine has not been extensively explored; hence, their impacts on wine flavor are still unclear [9,14].

Chardonnay is a white grape variety known as a neutral aromatic variety that produces wines without a peculiar set of aroma compounds [15]. It easily adjusts to many different terroirs in almost every wine-growing region [16]. Furthermore, the Chardonnay variety has gained popularity in wine-making regions because of its aging ability and its ease in reflecting the area where it is grown [17]. To the best of our knowledge, there are few reports on the use of purified *β*-glucosidase from natural yeasts to enhance the aroma of Chardonnay wines [18]. Therefore, in this study, we purified two *β*-glucosidases (Mg-βgl and Hu-βgl) isolated from the native non-*Saccharomyces* yeasts *Meyerozyma guilliermondii* NM218 and *Hanseniaspora uvarum* BF345 (chosen for their high *β*-glucosidase activity). The biochemical properties of both enzymes were studied and applied to the aging of Chardonnay young wines to determine whether they could be used to improve the aroma of the wines.

## 2. Materials and Methods

### 2.1. Yeast Strains and Media

Strains NM218 and BF345 were isolated from the wine region (China) and identified as *Meyerozyma guilliermondii* and *Hanseniaspora uvarum* through 26S rDNA D1/D2 sequencing. They have been uploaded to BLAST at NCBI under the identification code No. MW922835 and No. MW922834, respectively.

The strains NM218 and BF345 were cultured at 28 °C in yeast extract peptone dextrose (YPD) medium, consisting of 20 g/L glucose, 20 g/L peptone, and 10 g/L yeast extract. The *β*-glucosidase of strains NM218 and BF345 were obtained by incubation in medium (composed of 20 g/L glucose, 20 g/L peptone, 10 g/L yeast extract, 3 g/L NH_4_NO_3_, 4 g/L KH_2_PO_4_, 0.5 g/L MgSO_4_·7H_2_O, and 10 g/L tween 80) for 72 h at 28 °C in a tabletop thermostat shaker (HTHZ-82A, Shanghai Yuejin Medical Instrument Co., Ltd., Shanghai, China) set at 150 r/min.

### 2.2. β-Glucosidase Purification and Enzyme Activity Determination

The purification of *β*-glucosidase was performed by referring to the methods described by Cai et al. [19] and Baffi et al. [20], where enzyme extract was obtained as the supernatant after centrifugation of *β*-glucosidase production medium for 15 min at 4 °C and 7500× *g*. The enzyme extract was first added to 80% saturated ammonium sulfate, left overnight at 4 °C, then centrifuged (4 °C, 10,000 r/min, 10 min) using a desktop high-speed refrigerated centrifuge (H2050R, Changsha Xiangyi Centrifuge Instrument Co. Changsha, China), and the precipitate was collected and dissolved with an equal volume of citrate-phosphate buffer (20 mM, pH 5.0). This was then packed into dialysis bags (molecular weight cut off 50 kDa) for desalting by dialysis in sodium acetate buffer (50 mM, pH = 5.0) at 4 °C, and the dialysis solution was changed every 2 h. The desalting effect was detected by barium chloride. The desalted enzyme solution was concentrated to 2 mL by polyethylene glycol (PEG 20,000). Afterward, the concentrate was loaded on a DEAE-Sepharose-FF column pre-equilibrated 5 times the column volume of 20 mM sodium phosphate buffer at a pH of 5.0 until the baseline was smooth and the fractions were eluted with the gradient of 0–1 M NaCl at a flow rate of 1 mL/min. The fractions were collected into tubes for 5 min, and the *β*-glucosidase activity of each tube was determined. The fractions with higher enzyme activity were combined and concentrated again to 2 mL with PEG 20,000. Then, the concentrate was added to equilibrated acryldextran Sephacry1 S-200 chromatography column pre-equilibrated with the citrate-phosphate buffer (20 mM, pH 5.0), and the fractions were eluted with the gradient at a flow rate of 0.4 mL/min. After 5 min, the tubes were taken for the determination of enzyme activity. The highest enzyme activity fraction was the target *β*-glucosidases Mg-βgl and Hu-βgl. The resulting enzyme solution was stored at 4 °C. The *β*-glucosidase activity was determined under the conditions described by Hu et al. [21]. One unit (U) of enzyme activity was defined as the amount of enzyme released at 40 °C of 1 μmol *p*-nitrophenol/min.

### 2.3. Enzymatic Characterization

#### 2.3.1. Measurement of Optimal Temperature and pH Value

The optimum temperature for *β*-glucosidase activity was determined by incubating the purified enzymes Mg-βgl and Hu-βgl at different temperatures (10–70 °C). The optimal pH value of the purified enzymes Mg-βgl and Hu-βgl were determined at 40 °C in 20 mM citrate-phosphate buffer with a range of pH values (2.0–7.0). The *β*-glucosidase activities were measured according to the method described in Section 2.2.

#### 2.3.2. Effects of Metal Ions and Enzyme Inhibitors on Enzyme Activity

The effects of metal ions and enzyme inhibitors on enzyme activity were determined based on the method described by Mallek-Fakhfakh et al. [22]. The activities of Mg-βgl and Hu-βgl were evaluated in different metal ions (ZnSO_4_·7H_2_O, MgSO_4_·7H_2_O, MnSO_4_·H_2_O, FeSO_4_·7H_2_O, FeCl_3_, CuSO_4_·5H_2_O, CaCl_2_, CoSO_4_·5H_2_O, and Ni_2_SO_4_) at a final concentration of 5 mM at their optimum temperature and pH. The effects of enzyme inhibitors (dimethyl *β*-mercaptoethanol (β-ME), SDS, ethylenediaminetetraacetic acid (EDTA), and sulfoxide (DMSO)) on *β*-glucosidase activity were assessed at 1 mM final concentrations. The *β*-glucosidase activities were measured according to the method described in Section 2.2. Relative activity, defined as the ratio of enzyme activity in the presence of the test reagents or ions to that in the absence of enzyme inhibitors and metal ions, was also determined.

#### 2.3.3. Effect of Various Sugars on Enzyme Activity

The purified *β*-glucosidases Mg-βgl and Hu-βgl were preincubated for 30 min at optimum pH and temperature in the presence of various sugars (glucose, xylose, galactose, mannose, maltose, fructose, sucrose, arabinose, and cellobiose). Mg-βgl and Hu-βgl activities were evaluated at 0.1 M final concentrations of each sugar. The *β*-glucosidase activities were measured as described in Section 2.2. The ratio of enzyme activity in the presence of sugar to that in the absence of sugar defined the relative activity.

#### 2.3.4. Effect of Ethanol on Enzyme Activity

The effect of ethanol on enzyme activity was determined at optimum pH and temperature using various ethanol concentrations including 0%, 8%, 12%, and 15% *v/v* [23]. The 0% ethanol concentration was used as a control. The *β*-glucosidase activities were measured based on the method described in Section 2.2. Relative activity was defined as the ratio of enzyme activity in the presence of ethanol to that in the absence of ethanol.

### 2.4. Enzyme Treatment of Chardonnay Young Wines

Chardonnay grapes were harvested in 2020 from northwest China (Ningxia, China). The oenological parameters of the final Chardonnay must were as follows: pH = 3.53, total acidity = 6.87 g/L, and residual sugar = 249.79 g/L. The grapes were pressed directly, cold clarified, and the juice transferred to 5 L flasks with the addition of 50 mg/L sulfur dioxide (SO_2_), 20 mg/L pectinases, and 0.02 g/L *Saccharomyces cerevisiae* (Vintage Red^®^). Chardonnay must fermentation proceeded at 20 °C with daily stirring. The oenological parameters of the Chardonnay young wine were as follows: alcohol = 14.6% (*v/v*), volatile acids = 0.32 g/L, pH = 3.36, total acidity = 5.72 g/L, and residual sugar = 0.43 g/L. One enzymatic unit (U) of each purified *β*-glucosidase (Mg-βgl and Hu-βgl) was incubated in 50 mL Chardonnay young wine for 50 days [24]. Under the same conditions, control experiments with the addition of 1 U of commercial *β*-glucosidase from *Aspergillus niger* (An-βgl) and without enzyme were performed (Control, CK). All the experiments were performed (in triplicate) with or without purified enzymes at 18 °C under agitation.

### 2.5. Volatile Aroma Compounds Identification and Quantification

A slight modification to the method described by Wang et al. [25] was employed for volatile compounds determination using headspace solid-phase microextraction–gas chromatography-mass spectrometry (HS-SPME–GC–MS). The wine sample (8 mL) was added to a sample vial containing 2.5 g sodium chloride (NaCl) and 10 μL internal standard (2-octanol, with a concentration of 820.7 mg/L) and then equilibrated in a water bath at 40 °C for 30 min with stirring (20 r/min) [26]. A DVB/CAR/PDMS fiber (50/30 μm film thickness, Supelco, Bellefonte, PA, USA) coupled with a manual holder was immersed in the vial for 30 min with continuous stirring (20 rpm), followed by desorption into the GC injector for 5 min at 240 °C. Gas chromatography and mass spectrometry system (TRACE 1310-ISQ, Thermo Fisher Scientific, San Jose, CA, USA) equipped with a TG-WAX capillary column (60 m × 0.25 mm × 0.5 μm film thickness, Thermo) was used for the analysis of volatile compounds, and the carrier gas was helium (purity 99.999%) at a flow rate of 1 mL/min. The temperature of the GC oven started at 40 °C for 5 min, then was increased to 180 °C at 3.5 °C/min and maintained for 15 min. The MS transfer line and ion source temperatures were set at 180 °C and 250 °C, respectively. The ionization voltage (EI) was 70 eV, and the mass range was *m*/*z* 50–350.

Model wine solution (11% *v/v* ethanol, 6 g/L tartaric acid, and pH adjusted to 3.3–3.4 with 1 mol NaOH) used for obtaining standard calibration curves was made using standard chemical solutions as described by Tao et al. [27]. Supplementary Table S1 contains the qualitative and quantitative data for eleven chromatographically pure standards as well as standard calibration curves, R^2^ value, and linear range used for the quantification of the identified volatile compounds. Volatile compounds were identified according to the retention time and mass spectra of the pure standards compared with the NIST2.0 and Wiley databases (Thermo Scientific Software, San Jose, CA, USA). Standard calibration curves provided quantitative data for 11 volatiles, while the remaining 7 compounds were semi-quantified based on an internal standard (2-octanol).

### 2.6. Sensory Evaluation

A panel of 16 members, including staff and graduate students from Gansu Agricultural University (8 males and 8 females) with more than 2 years of experience in wine grading, conducted the wine sensory evaluation. Thirty milliliters (30 mL) of each wine sample were presented to the judges in random order at 18 °C. The wine samples were evaluated by each judge in an individual testing booth [24] using the triangle test method, where one cup of enzymatically treated wine was paired with two untreated wines. Mg-βgl-, Hu-βgl-, and An-βgl-treated wine samples were also evaluated using the triangulation test. Panelists were asked to evaluate samples by sniffing from left to right and to identify and describe the odd samples.

### 2.7. Data and Statistical Analysis

All experiments were assayed in triplicates. SPSS 19.0 software (SPSS, Inc., Chicago, IL, USA) was employed for data analysis using one-way analysis of variance (ANOVA). The significant differences were determined using Duncan tests at *p* < 0.05. Microsoft Excel 2016 (Microsoft, Redmond, WA, USA) and Origin 2018 (OriginLab, Inc., Northampton, MA, USA) were used to draw the figures.

## 3. Results

### 3.1. Purification of β-Glucosidase from M. guilliermondii NM218 and H. uvarum BF345

**Figure 1:**
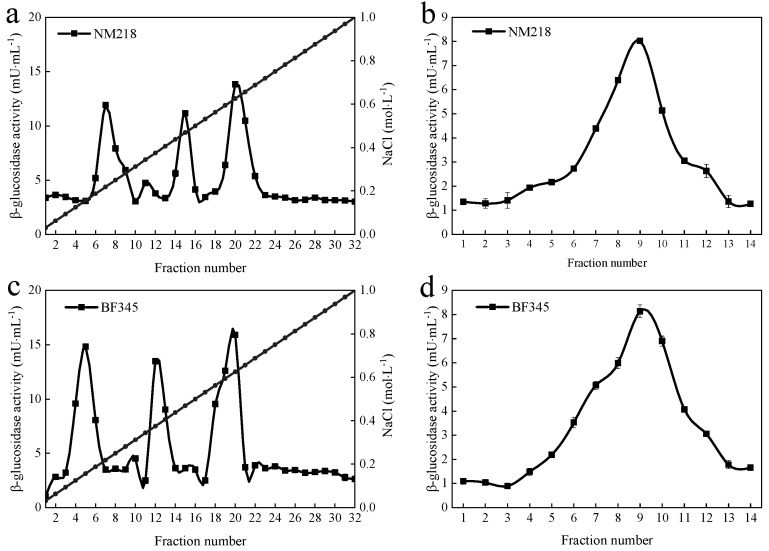
Purification of the *β*-glucosidase of *M. guilliermondii* NM218 and *H. uvarum* BF345. Fractions after DEAE-Sepharose-FF column ((**a**) for NM218 and (**c**) for BF345) containing high *β*-glucosidase activity. Resulting peak fractions containing high *β*-glucosidase activity purified on Sephacryl S−200 column ((**b**) for NM218 and (**d**) for BF345). In both columns, the collected high *β*-glucosidase activity fractions are those having more than 5 mU/mL. The curve represents the enzyme activity, and the line represents the NaCl concentration.

The purification protocol for the *β*-glucosidase of *M. guilliermondii* NM218 and *H. uvarum* BF345 is shown in Table 1. Compared with the enzyme extract, the *M. guilliermondii* NM218 enzyme was purified 12.2-fold with a specific enzyme activity of 1.95 U/mg. On the other hand, the *M. guilliermondii* BF345 enzyme was purified 12.4-fold with a specific enzyme activity of 2.11 U/mg. The extracellular extracts of strains *M. guilliermondii* NM218 and *H. uvarum* BF345 were precipitated by 80% ammonium sulfate solution, and the *β*-glucosidase obtained was further purified by chromatography columns. Throughout the elution process, a single peak with *β*-glucosidase activity was obtained on both DEAE-Sepharose-FF and Sephacry1 S-200 columns (Figure 1), indicating the presence of a single *β*-glucosidase.

### 3.2. Enzymatic Characterization

**Figure 2:**
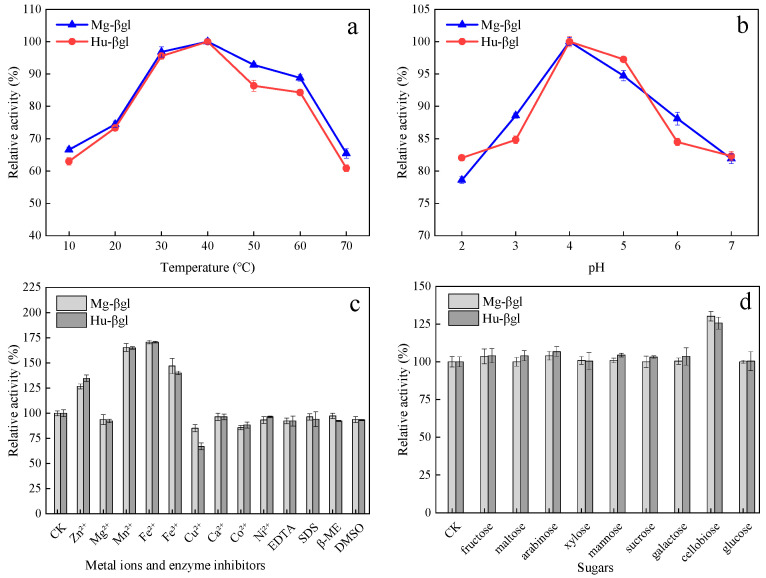
Temperature (**a**), pH (**b**), metal ions and enzyme inhibitors (**c**), and various sugars (**d**) profiles of the activity of the purified enzymes Mg-βgl and Hu-βgl. The values represent the mean of three replicate determinations. The error bars represent SD.

The effect of temperature, pH, metal ions, enzyme inhibitors, and various sugars on enzyme activity is shown in Figure 2a–d. The optimal catalytic temperature of Mg-βgl and Hu-βgl was 40 °C (Figure 2a). The relative activity of Mg-βgl and Hu-βgl remained at a high level (>70%) between 20 °C and 50 °C. At 60 °C or above, a significant decrease in enzyme activity was observed. The optimal catalytic pH of Mg-βgl and Hu-βgl are also shown in Figure 2b. Between a pH of 2.0 and 6.0, the enzyme activity of Mg-βgl and Hu-βgl was above 75%, indicating that Mg-βgl and Hu-βgl had an effective catalytic capacity in acidic medium. Furthermore, Mg-βgl and Hu-βgl had the highest activity at a buffer pH of 4.0.

Figure 2c shows the effect of metal ions and enzyme inhibitors on enzyme activity. The concentrations of Zn^2+^, Mn^2+^, Fe^2+^, and Fe^3+^ at 5 mM had a significant activating effect on the enzyme activity of the two enzymes Mg-βgl and Hu-βgl, contributing to an increase of 23.72–70.72%, while Cu^2+^ had a significant inhibiting effect on the enzyme activity of Mg-βgl and Hu-βgl, with a decrease of 14.87–32.89%. The four enzyme inhibitors selected in this experiment were inconspicuous for Mg-βgl and Hu-βgl, and the enzyme activity only decreased by 2.71–7.66%. The effect of various sugars on enzyme activity is presented in Figure 2d. The nine selected various sugars showed activating effects on Mg-βgl and Hu-βgl, among which cellobiose was particularly effective in activating the enzyme activity of Mg-βgl and Hu-βgl, increasing the enzyme activity by 25.67–40.80%. Besides cellobiose, other sugars did not show a significant activation effect on Mg-βgl and Hu-βgl.

**Figure 3:**
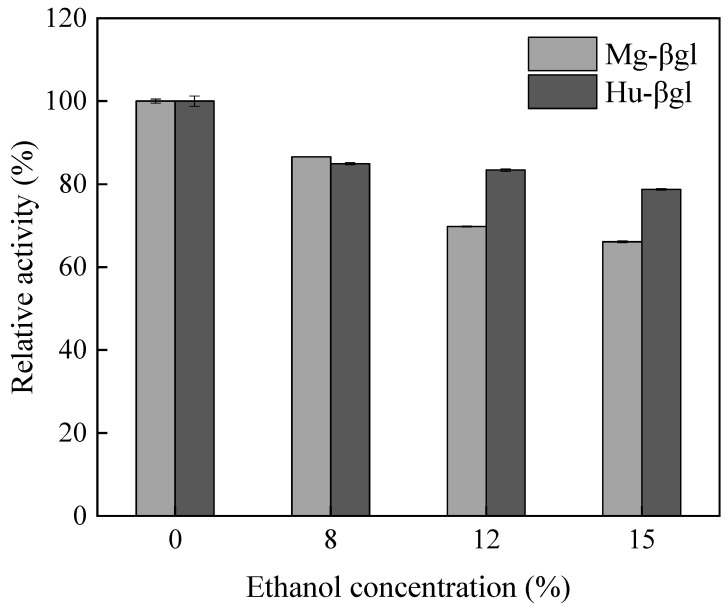
Effect of ethanol concentration on purified enzyme Mg-βgl and Hu-βgl. The values represent the mean of three replicate determinations. The error bars represent SD.

Regarding the effect of ethanol on enzyme activity, the activity of *β*-glucosidase was inhibited in the presence of ethanol (Figure 3). Mg-βgl maintained more than 65% activity at 15% ethanol concentration, while Hu-βgl maintained more than 75% activity at 15% ethanol concentration.

### 3.3. Analysis of Volatile Aroma Compounds in Chardonnay Wines

Table 2 compares the GC-MS results of volatile aroma components in purified Mg-βgl- and Hu-βgl-treated Chardonnay wines to An-βgl -treated and naturally aged wines. In this study, the activity of Mg-βgl and Hu-βgl released similar but low amounts of total terpenoids (0.033 mg/L and 0.034 mg/L, respectively) than the commercial enzyme An-βgl (0.039 mg/L). However, both enzymes significantly increased the concentration of volatiles in their treated wines compared with wines without *β*-glucosidase addition (CK). Between An-βgl-treated wines and purified Mg-βgl- and Hu-βgl-treated wines, there was no significant difference in the total terpenoids concentration, but regarding the concentrations of some specific monoterpene aglycones, significant differences were found. In particular, the activity of Mg-βgl and Hu-βgl resulted in significantly higher levels of linalool (0.023 mg/L each) than An-βgl (0.014 mg/L), and a 52% increase compared to CK. The three enzymes had a significant impact on the hydrolysis of glycosidically bound C_13_-norisoprenoids, resulting in higher concentrations of volatile aroma compounds than the control wine (CK), in which some volatiles were not detected. It is worth mentioning that the activity of An-βgl (0.034 mg/L) resulted in significantly higher total C_13_-norisoprenoids concentration (0.034 mg/L) than Mg-βgl and Hu-βgl (0.016 mg/L and 0.026 mg/L), and that geranyl acetone was only detected in An-βgl-treated wines. For C_6_ compounds, the lowest total concentrations were found in An-βgl-treated wines, while the activity of Hu-βgl resulted in the highest total concentrations of C_6_ compounds. Interestingly, hexanol showed the highest concentrations in Mg-βgl and Hu-βgl treatments, revealing the important role of *β*-glucosidase in hydrolyzing hexanol precursors. Thus, Mg-βgl and Hu-βgl were with a preference for hexanol and showed the highest concentrations, indicating the importance of *M. guilliermondii* NM218 and *H. uvarum* BF345 for *β*-glucosidase in hydrolyzing hexanol precursors.

After enzymatic treatments, the activity of Mg-βgl and Hu-βgl resulted in an increase in the total concentration of alcohols, esters, and fatty acids compared to CK, which contributed to the improvement of the aromatic complexity of the wines. Moreover, An-βgl treatment resulted in significantly lower total alcohols and esters but with remarkably higher total fatty acids compared with other treatments (Table 2). The alcohols were characterized by “vegetal” and “herbal” aromas. Among the alcohols, 2-phenylethanol showed the highest increase in Mg-βgl- and Hu-βgl-treated wines. The activity of Mg-βgl and Hu-βgl increased the concentration of 2-phenylethanol by 84% and 78%, respectively, compared to the control (CK). In addition, the significant increase in many esters (i.e., phenethyl acetate, ethyl octanoate, and ethyl heptanoate) in Mg-βgl- and Hu-βgl-treated wines suggests that *β*-glucosidase from *M. guilliermondii* NM218 and *H. uvarum* BF345 have a preference for their precursors. As a point of interest, the enzymatic activity of Hu-βgl increased the concentrations of ethyl octanoate, ethyl heptanoate, and ethyl caprate by 58%, 60%, and 72%, respectively, compared to the control (Table 2), which are known to impart floral and fruity aromas to wine and may positively contribute these to the aroma profile of the wines.

### 3.4. Sensory Evaluation of Wines

Panelists evaluated the wines to determine if differences existed in the aromas of Chardonnay wines treated with Mg-βgl, Hu-βgl, and An-βgl compared to the control (CK) wine. When a significant difference in the aroma was perceived, panelists were asked to freely record the descriptors. The evaluation showed that Mg-βgl, Hu-βgl, and An-βgl were significantly different from the control. The wines treated with Mg-βgl were perceived as sweet, floral, fruity, banana, and medicinal by the panelists, while Hu-βgl-treated wines were perceived sweeter with more floral, honey, pomelo, and banana aromas. In addition, wines treated with An-βgl were more sweet, floral, and fruity compared with CK (Table 3). These sensory descriptors perceived in wines treated with Mg-βgl and Hu-βgl correlated well with the previously observed GC-MS data (Table 2). When the panelists were asked to choose one of the wines among the three enzymatically treated wines, 94% preferred wines treated with Hu-βgl, indicating the potential of this enzyme as an aroma enhancer.

## 4. Discussion

The current competitive wine market demands increased typicality and differentiation of wines, and as a result, some wine-making methods allow the use of a variety of ancillary products or methods, such as the addition of enzymes to enhance wine aroma [35]. Most flavor precursors are not released during the wine-making process [36,37]. The addition of exogenous glycosidases to promote the hydrolysis of aroma glycosides is of interest to many winemakers. In particular, the enzyme from non-*Saccharomyces* yeasts has positive properties such as good tolerance to pH, temperature, or ethanol [38]. In this work, we aimed to characterize newly purified *β*-glucosidases of the native strains *M. guilliermondii* NM218 and *H. uvarum* BF345 isolated from grapes by focusing on the role and possible contribution of these enzymes in the improvement of young Chardonnay wine aroma.

The *β*-glucosidase was purified stepwise by ammonium sulfate precipitation, DEAE-Sepharose-FF column, and Sephacry1 S-200 column. Both Mg-βgl and Hu-βgl were purified 12-fold (Table 1 and Figure 1), and both enzymes showed high levels of specific activity (1.95 U/mg and 2.11 U/mg, respectively). Purified Mg-βgl and Hu-βgl exhibited high activity at pH 4.0 and were able to maintain more than 75% activity at pH 2.0, indicating that they are acid tolerant. This optimal pH is significantly different from the reported optimal pH of 5.0–6.0 for the *β*-glucosidase of *Pichia membranifaciens* Pm7, *Hanseniaspora vineae* Hv3, *H. uvarum* Hu8, and *Wickerhamomyces anomalus* Wa1, where only *H. uvarum* Hu8 maintained 50% activity at pH 3.0, and the other *β*-glucosidases lost most of their activity at pH 3.0 [13]. The optimal temperature recorded for both Mg-βgl and Hu-βgl was 40 °C, which was lower than the previously reported optimal temperature for *β*-glucosidases from *Pichia guilliermondii* G1.2 (55 °C) [39] and *Talaromyces thermophilus* (65 °C) [22]. The use of *β*-glucosidase, which can maintain activity at low temperatures and low pH, in industrial winemaking increases the solubility of reactants and products and contributes to the hydrolysis of biomass enzymatically [22]. The interaction between metal ions and enzymes can be found by identifying alterations in the secondary and/or tertiary structure of the protein that leads to reduced activity [40]. In this study, Zn^2+^, Mn^2+^, Fe^2+^, and Fe^3+^ had a significant activating effect, while the remaining metal ions had a certain inhibitory effect on the enzyme activity of Mg-βgl and Hu-βgl. This result suggests that these four ions can be further investigated and developed as activators of Mg-βgl and Hu-βgl. The influence of enzyme inhibitors (EDTA, SDS, β-ME, and DMSO) on the enzymes’ activity was also evaluated. They had an insignificant inhibitory effect on the enzyme activity of Mg-βgl and Hu-βgl, indicating that the enzymes still had catalytic ability amid these inhibitors. Interestingly, non-ionic detergents have been reported to improve the enzyme activity of some *β*-glucosidases, such as the *β*-glucosidase of *Fervidobacterium islandicum* [41]. Most *β*-glucosidase activities are susceptible to feedback inhibition by the product and require a high enzyme load to achieve a certain hydrolysis efficiency, which increases the cost of production [22]. Therefore, we assessed the impact of different sugars on the activity of these enzymes. The selected sugars showed activating effects on both Mg-βgl and Hu-βgl, especially cellobiose, which showed a significant activation. This is very beneficial to improve the hydrolysis efficiency of *β*-glucosidase. Additionally, Mg-βgl and Hu-βgl showed better tolerance to ethanol than most reported *β*-glucosidases of non-*Saccharomyces* yeast origin [9,42]. From some reported research, it appears that the alteration of some residues and the presence of specific motifs in the amino acid sequence could improve the tolerance of *β*-glucosidase to ethanol [43]. However, further investigation on the structure of the enzyme is needed to help researchers better understand this phenomenon.

During wine aging, the release of volatile compounds from their glycosides by acid catalysis is rather slow, except for the glycosides of allyl alcohol [44]. In contrast, exogenous glycosidases enable the rapid release of glycosides from sugar conjugates, which, due to the reactivity of the released glycosides in the wine pH, contributes to the accelerated synthesis of active compounds with volatile aromas during wine aging [44]. It has been reported that enzymatic treatment of wines followed by short-term bottle aging can help improve the aroma of wines [45], which is similar to the results of the present study. The results of this study showed that the use of Mg-βgl, Hu-βgl, and An-βgl in aging increased the total concentration of terpenes and C_13_-norisoprenoids. Codresi et al. [46] also reported an increase in the total concentration of terpenes and C_13_-norisoprenoids with the use of *β*-glucosidase at the end of alcoholic fermentation. Interestingly, *β*-glucosidase from *M. guilliermondii* NM218 and *H. uvarum* BF345 strongly increased the concentration of linalool compared to the control, whereas commercial *β*-glucosidase from *A. niger* showed an insignificant increment of linalool. This is in contrast to Castro et al. [47], who observed that treatment of Chardonnay wines with a commercial enzyme (AR-2000) increased linalool content by 64.2%. This suggests that *β*-glucosidases of different origins have different acting substrates and were more or less strongly influenced by the nature of the glycosidic elements [48]. In addition, geraniol was not detected in Mg-βgl- and Hu-βgl-treated wines, but it was detected in An-βgl-treated wines. However, its concentration did not exceed the odor perception threshold (OAV < 1), a result that can be attributed to the presence of a small number of precursors in this wine. Meanwhile, this study found that C_13_-norisoprenoids were detected only in enzymatically treated wines. Mateo et al. also reported that enzyme treatment increased C_13_-norisoprenoids concentrations from 265 to 2000% [49]. C_13_-norisoprenoids are mainly glycosylated in grapes, and unlike terpenes, they are present in nearly equal amounts in all varieties. Because they have a lower odor threshold than terpenes, they can provide aromatic characteristics that confer certain typicality to wine flavors when hydrolyzed [49,50]. C_6_ compounds detected in wines generally impart raw green or herbal flavors to wines [31], but high concentrations of C_6_ compounds above their thresholds impart undesirable grassy flavors to wines [51]. In this study, none of the C_6_ compounds detected exceeded their odor thresholds. Notably, hexanol showed the highest concentration in Mg-βgl- and Hu-βgl-treated wines, which reveals the important role of *β*-glucosidase in the hydrolysis of hexanol precursors. The aglycon fraction is usually formed by terpenols, C_13_-norisoprenoids, and C_6_ compounds. Other aroma precursors can also occur, such as alcohols, phenolic acids, and possibly volatile phenols [50]. In terms of other aroma components, the wines treated with Mg-βgl and Hu-βgl had a higher content of higher alcohols, ethyl esters, and fatty acids compared to the control (CK) wines. However, this was not observed in the wines treated with An-βgl. This may be due to the different sources of *β*-glucosidase [52]. There were no significant differences in the total amount of alcohols and fatty acids in the Mg-βgl- and Hu-βgl-treated wines, indicating that the enzymatic characterization of Mg-βgl and Hu-βgl are similar, and as described above, their *β*-glucosidases may have similar mechanisms of action. Therefore, specific mechanisms of action and structures of Mg-βgl and Hu-βgl needs further investigation in the future. Higher alcohols and ethyl esters can impart a distinct fruitiness to the wines. It has been found that in Chardonnay wines, fruity and floral characteristics correlate with the number of compounds such as phenethyl acetate [3]. The concentration of phenethyl acetate was above the odor threshold in all wines, especially in Mg-βgl-treated wines, where the concentration of phenethyl acetate was significantly higher than in other wines. Additionally, enzymatic treatment increased the content of fatty acids in the wines. Although fatty acids tend to negatively affect the aroma of wines [53], they are involved in the equilibrium reactions during the formation of the corresponding esters. Therefore, increasing the concentration of fatty acids can enhance the synthesis of the corresponding esters [54]. In sensory evaluation, the aromas of the wines treated with Mg-βgl were perceived as sweet, floral, fruity, banana, and medicinal. Those treated with Hu-βgl were sweeter and fruitier, with more floral, honey, grapefruit, and banana aromas, while those treated with commercial *β*-glucosidase were more sweet, floral, and fruity. This result also validates the above data analysis.

Thus, short-term aging of Chardonnay wines after enzymatic treatment can lead to aroma enhancement. This work facilitates the use of exogenous glycosidases in the wine-making process to enhance the flavor of wines. However, further studies on the construction of yeast strains expressing the proteins involved in the release of flavor need to be conducted by genetic engineering techniques to enable the production of modified glycosidases without undesirable activity, intending to use for large-scale winery production.

## 5. Conclusions

In conclusion, purified *β*-glucosidases (Mg-βgl and Hu-βgl) from non-*Saccharomyces* yeasts *M. guilliermondii* NM218 and *H. uvarum* BF345 exhibited better enzymatic characterization and were applicable in Chardonnay wine fermentation and aging. The use of Mg-βgl and Hu-βgl during the aging process helped to improve the number and concentrations of the wine’s aroma compounds. Both enzymes significantly contributed esters, terpenes, C_13_-norisoprenoids, higher alcohols, and fatty acids to the treated wines, which enriched the aromatic complexity of the wines. Among them, *β*-glucosidase treatment from *M. guilliermondii* NM218 (Mg-βgl) imparted more sweet, floral, fruity, and banana aromas to the wine, whereas *β*-glucosidase treatment from *H. uvarum* BF345 (Hu-βgl) imparted sweeter, floral, honey, pomelo, and banana aromas to the wine.

## Figures and Tables

**Table 1:** Purification protocol for the *β*-glucosidase from *M. guilliermondii* NM218 and *H. uvarum* BF345.

Purification Steps	Total Activity/(U)	Total Protein/(mg)	Specific Activity/(U·mg^−1^)	Purification/(Fold)
*M. guilliermondii* NM218				
Enzymatic extract	22.41	138.61	0.16	1
80% ammonium sulfate	17.46	59.37	0.29	1.8
DEAE-Sepharose-FF	3.54	10.43	0.34	2.1
Sephacry1 S-200	1.77	0.91	1.95	12.2
*H. uvarum* BF345				
Enzymatic extract	24.68	149.2	0.17	1
80% ammonium sulfate	19.06	65.76	0.29	1.7
DEAE-Sepharose-FF	4.62	10.32	0.45	2.7
Sephacry1 S-200	1.86	0.88	2.11	12.4

**Table 2:** Volatile aroma compounds of Chardonnay wines treated with purified enzymes (Mg-βgl and Hu-βgl) and An-βgl.

Aroma Compound	Compound Concentration (mg/L)				Threshold (mg/L)	Odor Description
	Hu-βgl	Mg-βgl	An-βgl	CK		
Terpenes						
Geraniol	NA	NA	0.010 ± 0.002	NA	0.02 [28]	Lemon, peach, rose
Citronellol	0.010 ± 0.000 b	0.011 ± 0.001 b	0.015 ± 0.003 b	0.008 ± 0.001 a	0.03 [29]	Grassy, lilac, rose
Linalool	0.023 ± 0.001 b	0.023 ± 0.001 b	0.014 ± 0.001 a	0.012 ± 0.001 a	0.015 [30]	Rose, citrus, fruity, sweet
Total	0.033 ± 0.001 b	0.034 ± 0.002 b	0.039 ± 0.006 b	0.020 ± 0.002 a		
C_13_-norisoprenoids						
Damastone	0.016 ± 0.001 a	0.026 ± 0.001 b	0.030 ± 0.007 b	NA	0.05 [28]	Bark, canned peaches, baked apples, plums
Geranyl acetone	NA	NA	0.004 ± 0.001	NA	0.06 [31]	Light sweet fragrance, rose
Total	0.016 ± 0.001 a	0.026 ± 0.001 b	0.034 ± 0.008 c			
C_6_ Compounds						
Hexanol	0.116 ± 0.004 b	0.095 ± 0.005 b	0.070 ± 0.005 a	0.074 ± 0.002 a	8 [32]	Grass
2-Ethylhexanol	0.024 ± 0.006 c	0.013 ± 0.004 b	0.004 ± 0.000 a	0.010 ± 0.003 b		
Total	0.140 ± 0.010 c	0.108 ± 0.009 b	0.074 ± 0.005 a	0.084 ± 0.005 b		
Alcohols						
Benzyl alcohol	NA	NA	0.005 ± 0	NA	200 [32]	Toasted, fruity
2-Phenylethanol	5.412 ± 0.458 b	5.810 ± 0.650 b	4.122 ± 0.567 a	4.568 ± 0.531 a	1.4 [33]	Rose, honey
Isobutanol	0.051 ± 0.002 c	0.043 ± 0.003 b	0.028 ± 0.003 a	0.042 ± 0.002 b	40 [34]	Solvent, raw green
Total	5.463 ± 0.460 c	5.853 ± 0.653 c	4.155 ± 0.570 a	4.610 ± 0.533 b		
Esters						
Phenethyl acetate	0.608 ± 0.07 a	0.738 ± 0.03 c	0.605 ± 0.12 a	0.696 ± 0.052 b	0.25 [32]	Rose, jasmine
Ethyl caproate	0.555 ± 0.054 b	0.503 ± 0.038 a	0.539 ± 0.017 ab	0.571 ± 0.021 b	0.014 [34]	Banana, green apple, strawberry, anise
Ethyl octanoate	2.130 ± 0.193 d	1.482 ± 0.132 c	0.008 ± 0.002 a	1.226 ± 0.042 b	0.005 [25]	Rose fragrance, neroli oil, cool fruity
Ethyl heptanoate	0.093 ± 0.009 d	0.019 ± 0.001 b	0.003 ± 0.000 a	0.056 ± 0.002 c	0.22 [31]	Banana, green apple, strawberry
Ethyl caprate	0.723 ± 0.166 d	0.380 ± 0.014 b	0.118 ± 0.019 a	0.523 ± 0.029 c	0.2 [31]	Coconut fruit
Ethyl butyrate	0.031 ± 0.003 a	0.024 ± 0.003 a	0.020 ± 0.001 a	0.031 ± 0.001 a	0.02 [32]	Banana, pineapple, strawberry
Total	4.140 ± 0.495 c	3.146 ± 0.218 b	1.293 ± 0.159 a	3.103 ± 0.147 b		
Fatty acids						
Hexanoic acid	0.106 ± 0.009 b	0.092 ± 0.006 b	0.069 ± 0.046 a	0.055 ± 0.039 a	0.42 [32]	Fatty, cheesy
Octanoic acid	0.046 ± 0.005 a	0.059 ± 0.024 a	0.105 ± 0.029 c	0.072 ± 0.046 b	0.5 [33]	Putrid, pungent, cheesy
Total	0.152 ± 0.014 b	0.151 ± 0.004 b	0.174 ± 0.023 c	0.127 ± 0.043 a		

“NA” indicates not detected. Data are means ± standard deviation of three replicates. Data with different superscript letters (a,b,c,d) within each row are significantly different (Duncan tests; *p* < 0.05).

**Table 3:** Sensory analysis of Chardonnay wine treated with purified enzymes (Mg-βgl and Hu-βgl) and commercial enzyme (An-βgl).

Triangle Test	Number of Trials	Number of Correct Answers	Significance Level	Odor Descriptors
Mg-βgl vs. CK	16	14	*p* ≤ 0.001	Sweet, floral, fruity, banana, medicinal
Hu-βgl vs. CK	16	14	*p* ≤ 0.001	Sweet, floral, honey, pomelo, banana
An-βgl vs. CK	16	12	*p* ≤ 0.001	Sweet, floral, fruity
Mg-βgl vs. An-βgl	16	13	*p* ≤ 0.001	Sweet, floral, fruity, banana
Hu-βgl vs. An-βgl	16	13	*p* ≤ 0.001	Floral, fruity, toasty

## Data Availability

The data presented in this study are available on request from the corresponding author.

## Supplementary Materials

The following is available online at https://www.mdpi.com/article/10.3390/foods11060852/s1, Table S1: Qualitative and quantitative information of twenty-six chromatographically pure standards and their calibration curves, R^2^ values, and linear ranges.
