# Supplementary material for: Enzymatic Characterization of Purified β-Glucosidase from Non-Saccharomyces Yeasts and Application on Chardonnay Aging
Source: Foods. 2022 Mar 17;11(6):852. doi: 10.3390/foods11060852 (PMC8950599; doi:10.3390/foods11060852)
Supplement: Supplementary file 1 [file foods-11-00852-s001.zip › foods-1625967-supplementary.pdf]

foods

MDPI

Supplementary Table S1 Qualitative and quantitative information of eleven chromatographically pure standards and their calibration curves, R^2 values, and linear ranges.

**Compounds**	**Purity**	**CAS in Sigma**	**CAS**	**Density**	**Linear rang (mg/L)**	**Calibration curves**	**R^2 value**
Benzyl alcohol	99.8%(GC)	305197-100ML	100-51-6	1.045 g/mL at 25 °C	0.13-1.96	$y = 2\times10^7x - 7\times10^5$	0.9900
Ethyl heptanoate	99.0%(GC)	112364-100ML	106-30-9	0.870 g/mL at 25 °C	0.01-3.26	$y = 2\times10^9x + 1\times10^8$	0.9907
Hexanol	≥99.9%(GC)	73117-1ML-F	111-27-3	0.814 g/mL at 25 °C	0.01-1.53	$y = 4\times10^7x + 2\times10^6$	0.9982
Hexanoic acid	≥99.0%(GC)	21529-5ML	142-62-1	0.927 g/mL at 25 °C	0.29-4.64	$y = 5\times10^6x + 1\times10^6$	0.9938
Octanoic acid	99.5%(GC)	21639-5ML	124-07-2	0.910 g/mL at 25 °C	0.11-3.41	$y = 5\times10^6x + 8\times10^5$	0.9731
Ethyl butyrate	99.0%(GC)	E15701-500ML	105-54-4	0.875 g/mL at 25 °C	0.02-2.19	$y = 6\times10^7x + 3\times10^6$	0.9970
Ethyl caproate	99.0%(GC)	148970-100ML	110-38-3	0.862 g/mL at 25 °C	0.11-4.31	$y = 3\times10^9x - 2\times10^8$	0.9880
2-Phenylethanol	99.0%(GC)	77861-250ML	60-12-8	1.020 g/mL at 20°C	0.26-15.30	$y = 3\times10^7x + 2\times10^7$	0.9893
Phenethyl acetate	97.0%(GC)	W285706-SAMPLE-K	103-45-7	1.032 g/mL at 25 °C	0.01-1.94	$y = 5\times10^8x + 5\times10^6$	0.9932
Citronellol	95.0%(GC)	W230901-SAMPLE-K	106-22-9	0.855 g/mL at 25 °C	0.11-2.14	$y = 3\times10^6x + 1\times10^6$	0.9963
Damascenone	≥90.0%(GC)	30395-1ML	23726-91-2	0.934 g/mL at 20 °C	0.06-3.50	$y = 3\times10^7x + 2\times10^6$	0.9994
2-octanol	99.5%(GC)	74858-25mL	123-96-6	0.819 g/mL at 20 °C	/	/	/
